# Variations in efficiency of plastidial RNA editing within *ndh* transcripts of perennial ryegrass (*Lolium perenne*) are not linked to differences in drought tolerance

**DOI:** 10.1093/aobpla/plt035

**Published:** 2013-08-13

**Authors:** Rob J. M. Van Den Bekerom, Philip J. Dix, Kerstin Diekmann, Susanne Barth

**Affiliations:** 1 Teagasc Crops, Environment and Land Use Programme, Oak Park Crops Research Centre, Carlow, Ireland; 2 National University of Maynooth, Maynooth, Co. Kildare, Ireland

**Keywords:** Drought stress, *Lolium perenne*, NDH complex, plastidial RNA editing.

## Abstract

Maintenance of healthy grasslands is essential for efficient livestock production, yet projected climate change is likely to place a heavy drought stress burden on key grassland species, such as perennial ryegrass (*Lolium perenne*). It is therefore important to gather an in-depth knowledge of the underlying plant response to this stress. The present study is focused on RNA editing (post-transcriptional nucleotide modifications resulting in altered transcripts) within plastidial transcripts of the NADH:ubiquinone oxidoreductase (NDH) complex (NADH dehydrogenase complex) in relation to the drought response of several accessions of perennial ryegrass. Previous studies have shown that the NDH complex is involved in countering oxidative stress during environmental stresses like drought. Owing to the nature of RNA editing within this complex, the RNA editing machinery could play a potential role in regulating the activity of the NDH complex. The investigation revealed dramatic and reproducible differences in RNA editing efficiency between accessions, but efficiency was not influenced by imposition of drought stress, and a direct relationship between editing behaviour and drought response was not detected.

## Abstract (precis)

Projected climate change is likely to subject key temperate grassland species, such as perennial ryegrass (Lolium perenne) to drought stress. Previous studies have shown that the NADH dehydrogenase complex (NDH) is involved with countering oxidative stress during environmental stresses like drought. We studied RNA editing within plastidial transcripts of the NDH complex in relation to the drought response of several accessions of perennial ryegrass. We found dramatic and reproducible differences in RNA editing efficiency between accessions, but efficiency was not influenced by imposition of drought stress, and a direct relationship between editing behaviour and drought response was not detected.

## Introduction

Maintenance of healthy grasslands is essential for efficient livestock production, yet projected climate change is likely to place a heavy drought stress burden on key grassland species, such as perennial ryegrass (*Lolium perenne*). Perennial ryegrass-dominated pasture is the basis for livestock production in many temperate regions. However, as a consequence of estimated climate change over the next 100 years, the viability of the production of forage grasses will be threatened due to changes in temperature and rainfall (Holden and Brereton 2002). By improving the drought tolerance of cultivars of perennial ryegrass, the impact of climate change can be countered. This could be accomplished by either traditional breeding or genetic engineering, both of which will benefit from a deeper understanding of the underlying basis of drought stress responses in these crops.

The finding (Shinozaki *et al.* 1986) that the chloroplast genome includes close homologues to the genes encoding the subunits of the mitochondrial NADH:ubiquinone oxidoreductase (NDH) posed questions on the role of this complex in chloroplasts, which were resolved only when the availability of chloroplast transformation procedures allowed the characterization of gene knockouts (Burrows *et al.* 1998; Horváth *et al.* 2000; Joët *et al.* 2001). These confirmed that the complex is fully functional but is non-essential under normal growth conditions (Burrows *et al.* 1998), and suggested an adaptive role in relation to drought stress (Horváth *et al.* 2000). Specifically, when the *ndhB* gene was inactivated in transplastomic plants, the dark reduction in the plastoquinone (PQ) pool was impaired, and enhanced growth retardation was observed under humidity stress conditions (Horváth *et al.* 2000). Support for this role comes from the finding that NDH complex activity in thylakoids increases under a combination of drought, high light and temperature stress (Ibanez *et al.* 2010). Sequencing of the perennial ryegrass plastome revealed that genes encoding NDH proteins appear to be a hot spot for RNA editing sites, with more than half the detected editing sites being located in these genes (Diekmann *et al.* 2009). RNA editing alters the nucleotide sequence of an RNA molecule so that it deviates from the sequence of its DNA template and provides a novel post-transcriptional regulatory system in organelles (Tillich *et al.* 2006). Different RNA editing systems exist and each is thought to have evolved independently (Gray 2012). For example, individual pentatricopeptide repeat (PPR) proteins have recently been implicated in editing at specific sites in the *ndh* complexes of both mitochondria (Yuan and Liu 2012) and chloroplasts (Okuda *et al.* 2010). RNA editing in chloroplasts belongs to the conversion system, where exclusively C-to-U substitutions occur, with the exception of U-to-C substitutions in the bryophyte *Anthoceros formosae* (Kugita *et al.* 2003). mRNA editing usually results in the restoration of codons for conserved amino acids (Bock *et al.* 1994). It is plausible that the functionality of the NDH complex could be impaired by the lack of RNA editing, thereby decreasing the tolerance to oxidative stress caused by water deficit. This would imply that the RNA editing machinery could act as a ‘switch’ to turn on defences when exposed to stress conditions. To evaluate the potential of RNA editing efficiency as a marker for stress tolerance, or as a target for genetic modification, the current investigation sought to establish whether there is a correlation between the editing efficiency of *ndh* genes and drought tolerance, in a number of *L. perenne* accessions.

## Methods

### Plant material

*Lolium perenne* cultivar ‘Cashel’ was obtained from Teagasc, Oak Park Research Centre, Carlow, Ireland. Eight other accessions were acquired from the Germplasm Resources Information Network (http://www.ars-grin.gov/npgs/acc/acc_queries.html) in the USA, with different countries of origin: PI462336, New Zealand; PI231565, Libya; PI610825, Switzerland; PI418701, Former Yugoslavia; PI632553, Italy; PI611044, Russia; PI201187, The Netherlands; PI223178, Greece.

### Plant culture conditions and drought treatment

At least 12 plants of each accession were allowed to establish in a hydroponic system supplemented with 4.4 g L^−1^ Gamborg B5 medium + vitamins (Gamborg *et al.* 1968). The system was aerated by an aquatic pump to supply oxygen in the solution. Two separate systems were set up. After 1 week, the solution was refreshed to prevent depletion of nutrients. In the second week after experimental set-up, the drought stress experiment was initiated by replacing the solutions in both systems. In the first system the solution was replaced with 4.4 g L^−1^ MS (Murashige and Skoog 1962) salts; this system acted as the control. In the second system, the solution was replaced with a solution comprising 4.4 g L^−1^ MS salts supplemented with 20 % PEG-6000 (Duchefa cat. no. P0805) for the induction of drought stress. This concentration of PEG results in a water potential of about −0.45 MPa (Michel and Kaufmann 1973). The experiment was performed in a controlled glass house at Teagasc, Oak Park, Carlow, Ireland, with a mean daily temperature of 22 °C and supplemented with lighting (photosynthetically active radiation = 650 µE m^−2^ s^−1^) for 16 h. After 2 weeks in these conditions, samples were taken for analysis.

### Phenotypic growth assessment using pixel detection

**Figure 1.**
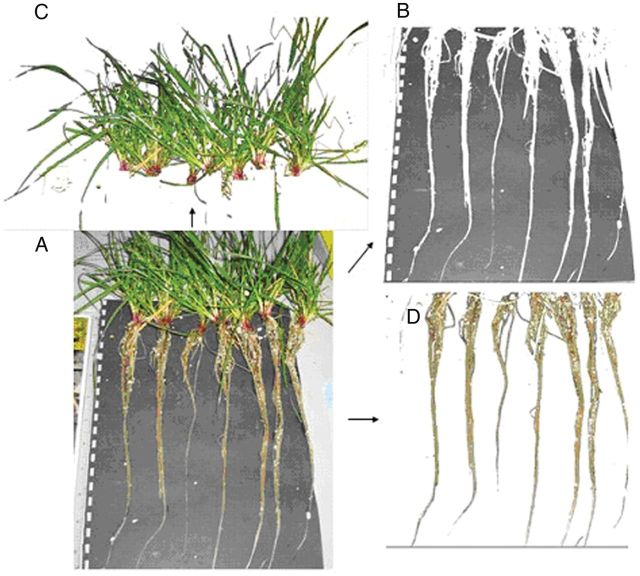
Analyses of amounts of different tissues using pixel detection. (A) Complete photograph. (B) Pixels consisting of background. (C) Pixels consisting of leaf tissue. (D) Pixels consisting of root tissue.

Photographs were taken of the plants after the completion of the experiment and analysed using Adobe Photoshop 5.5. Pixel detection of leaf tissue and root tissue was performed (Fig. 1), revealing the number of pixels in each photograph consisting of either leaf (Fig. 1C) or root tissue (Fig. 1D), thereby indicating the amount of tissue present after each treatment. Data from individual photographs were converted using a reference area in each photograph to detect the number of pixels therein (Fig. 1B), and the ratio of pixels between each photograph for this reference area represented the difference in the size of the photograph. A measure of relative biomass growth is represented by the ratio of pixels between treatments/accessions. The number of pixels within leaf tissue of individual plants was predicted by taking the total number of pixels consisting of leaf tissue divided by the number of plants in the photograph. For pixel detection of root tissue, individual plants could be assessed, since root tissue did not overlap between separate clones.

### Relative water content measurements

Relative water content (RWC) was measured as described previously (Barrs and Weatherley 1962). It was calculated for each plant by taking a 2-cm leaf piece from the middle of the plant and weighing the fresh weight (FW), turgor weight (TW) and dry weight (DW) of these leaf tissues. The tissues were submerged in distilled water for 3 h, after which the seedlings were blotted dry. For these seedlings, the TW was measured on a fine scale. Subsequently, the seedlings were placed in an oven to dry at 70 °C, after which the DW was measured.

RWC is calculated using the formula:
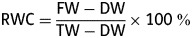



### Total dry biomass measurements

The total roots from each plant were harvested, wrapped in tin foil and dried in an oven at 70 °C for 3 days. The total root DW was recorded afterwards for each separate plant. The leaf DW could not be recorded, as the total leaf tissue was required to extract total RNA for RNA editing analyses.

### cDNA preparations

Total RNA from three plants per treatment and per accession was extracted using the RNeasy^®^ Plant Mini Kit from Qiagen (cat. no. 74903). mRNA extraction was performed using the manufacturer's instructions. During the RNA extraction, DNase was added. cDNA was synthesized from the RNA template using Superscript III reverse transcriptase following the manufacturer's instructions (Invitrogen cat. no. 18080-400).

### Polymerase chain reaction to amplify *ndhB* and *ndhF* fragments from cDNA and genomic DNA

Primer sequences were deduced from the published sequence (Diekmann *et al.* 2009) of the *L. perenne* plastome. For the *ndhB* fragment primer set 9 (P9 fwd: tccttcgtagacgtcag, P9 rev: ttggatgcagttactaattc) was used, while for the *ndhF* fragment primer set 88 (P88 fwd: ggagctagtaaccaatccca, P88 rev: agtaaaaattgcaatttcttttc) was used. Fragments were amplified using GoTaq polymerase (Promega cat. no. M8301) according to the manufacturer's instructions. Polymerase chain reaction (PCR) conditions were 35 cycles of 95 °C (1 min), 45 °C (1 min), 72 °C (1 min), followed by an extension step at 72 °C (10 min). The products were stored at 4 °C.

### Trace-file method to identify the editing efficiency

**Figure 2.**
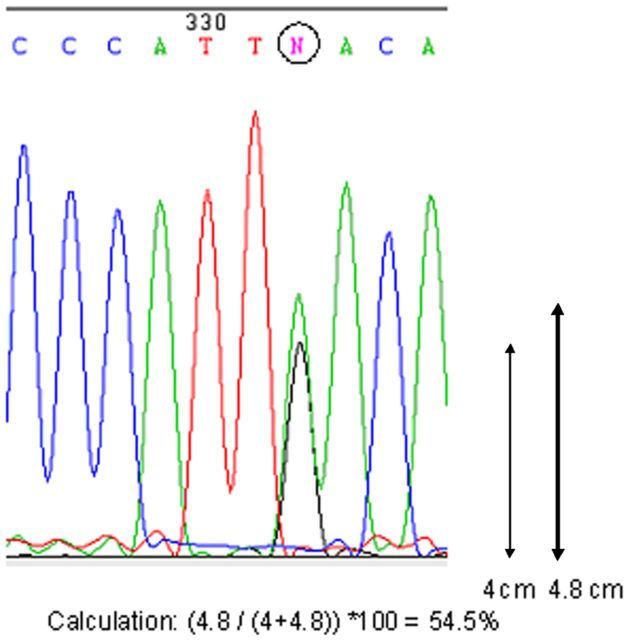
Calculation of editing efficiency using the trace-file method. The circle around the nucleotide represents the editing site.

All PCR products were sent to a commercial sequencing company for PCR purification and sequencing. The returned trace files were analysed with the program ‘Chromas Lite’ for RNA editing sites and the corresponding efficiency. To analyse the efficiency of editing, the heights of the peaks at an editing location within the trace files were measured and compared with one another as illustrated in Fig. 2. The editing efficiency was calculated using the formula (Nakae *et al.* 2008):
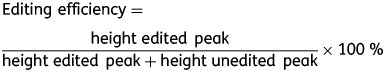

### Verification of trace-file methodology by comparison with a colony screen

Polymerase chain reaction products of the *ndhB* and one *ndhF* fragment were sequenced. Peaks in the resulting trace files were analysed using the formula stated above. The same PCR products were cloned into the cloning vector pCR2.1-TOPO, and subsequently introduced into *Escherichia coli* strain TOP10. Agar stab cultures were made in 96-well plates, each well containing a separate clone, derived from *E. coli* with pCR2.1-*ndhB* and *E. coli with* pCR2.1*-ndhF*. The clones containing the *ndhB* fragment were sequenced in both directions, whereas the clones containing the *ndhF* fragment were sequenced only in one direction. The editing efficiency was calculated as the percentage of clones containing the edited site, in comparison with the total number of clones. Colony screen results were compared with those of the trace-file method for quality assurance purposes.

Peptide alignments of *ndhB* and *ndhF* were made in CLC Sequence Viewer.

### Statistical analyses

The arcsine transformation and *t*-tests were conducted in Microsoft Excel, whereas the variance tests were performed in the program Minitab Solutions 15. All data sets were analysed for equal variance using the Levene test to determine whether *t*-tests were performed with equal or unequal variance. For the RWC analyses, arcsine transformation of the values was necessary to obtain a normal distribution to carry out statistical analyses, as percentage values cannot be used directly for comparison studies. These values were subsequently tested for statistical differences using the *t*-test with a one-tailed distribution and equal variance. A one-tailed distribution was chosen, as the hypothesis was that stressed plants would exhibit lower values compared with plants in control conditions.

For the total DW analyses, the results were tested for a normal distribution and subsequently tested for statistical differences using the *t*-test with a two-tailed distribution and unequal variance. A two-tailed distribution was chosen, based on the hypothesis that the root biomass could be either higher or lower for the stressed conditions compared with the control conditions.

For editing efficiency, the values were transformed into arcsine values. These values were subsequently tested for statistical differences using the *t*-test with a two-tailed distribution and unequal variance. A two-tailed distribution was chosen, based on the hypothesis that efficiency could be either lower or higher for plants under stressed conditions compared with plants in control conditions.

## Results

### Characterization of plant growth, evaluated after a drought stress period

**Figure 3.**
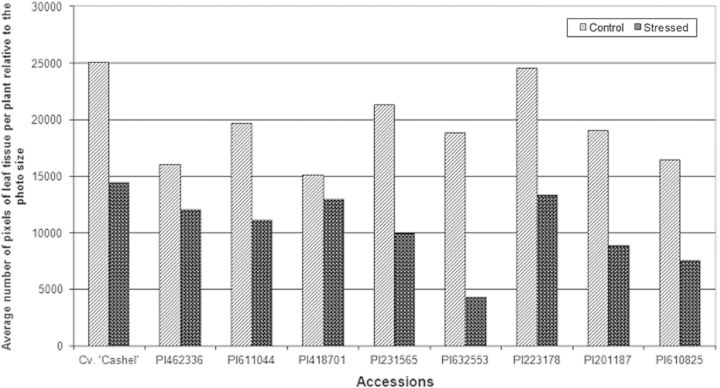
Relative average leaf biomass per plant, derived from total leaf biomass, based on pixel detection per accession. (No statistical analyses could be performed due to relative values.)

The average leaf biomass (Fig. 3) of accessions PI418701 and PI462336 was slightly decreased in leaf development under stress conditions, indicating a drought-tolerant response for clones within these accessions. All the other accessions had a clear decrease in biomass production under stress conditions, indicating a drought-susceptible response (Foito *et al.* 2009).

**Figure 4.**
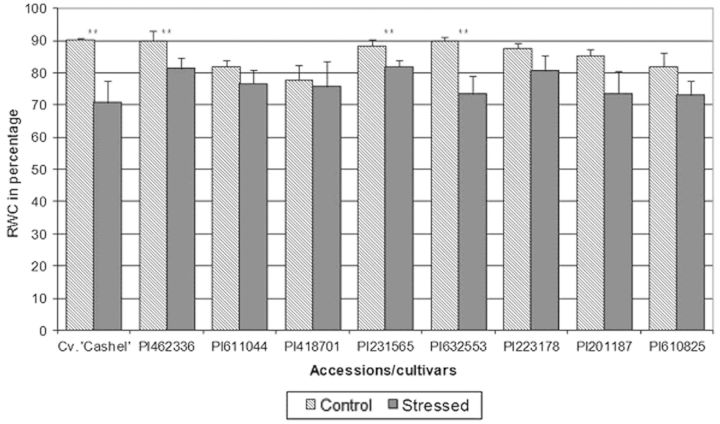
Relative water content after 2 weeks of exposure to drought stress compared with control conditions. Error bars represent the standard deviation of the mean. **Statistical difference between stress and non-stress treatments according to a *t*-test (one-tailed distribution, equal variance on arcsine transformed values) at *P* < 0.05.

There was a statistically significant difference (*P* < 0.05) in RWC between stressed and non-stressed plants for Cv. ‘Cashel’, and accessions PI462336, PI231565 and PI632553 (Fig. 4), indicating that plants from these accessions were susceptible to drought stress.

**Figure 5.**
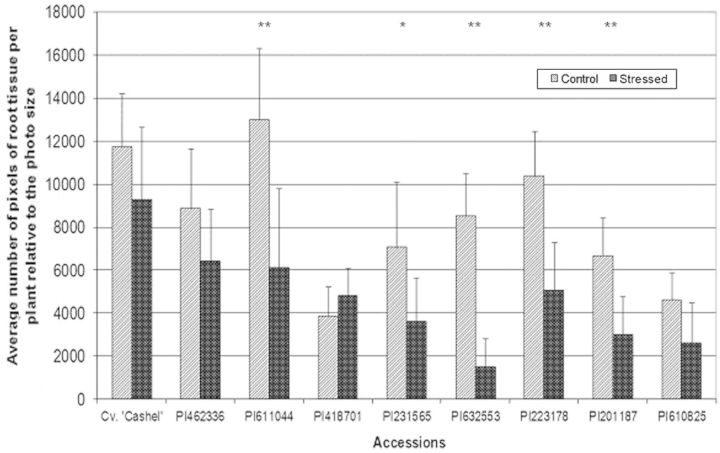
Mean root biomass per plant based on pixel detection. Statistical differences in the number of pixels between stress and non-stress treatments were calculated according to the *t*-test (two-tailed distribution, unequal variance). Error bars represent the standard deviation of the mean. Statistical differences at **P < 0.01, and *P < 0.05.

**Figure 6.**
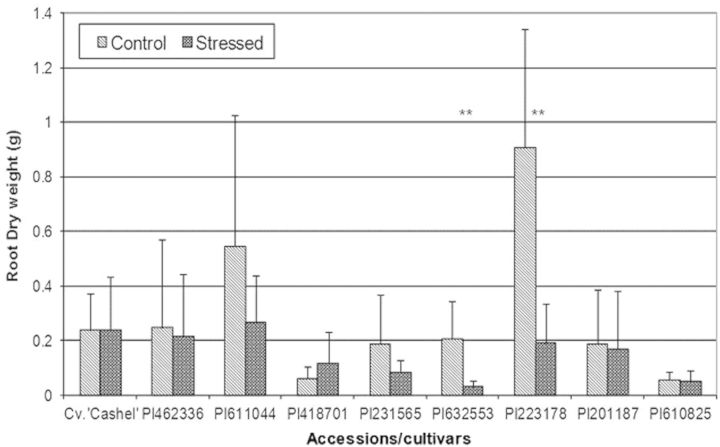
Mean root dry biomass per accession. Error bars represent the standard deviation of the mean. **Accessions, which had a statistical difference in dry root biomass (*t*-test, two-tailed distribution, unequal variance *P* < 0.05) between stress and non-stress conditions.

The increase in root biomass under drought stress reflects an adaptive response involving an increase in root length to reach water deeper in the soil (van den Berg and Zeng 2006). Accessions PI611044, PI632553, PI223178, PI201187 and PI231565 showed a significant decrease in root development after exposure to drought stress compared with control conditions, indicating that these accessions are drought susceptible (Fig. 5). Accession PI418701 exhibited a slight increase in root growth during drought stress compared with controll conditions, suggesting a drought-tolerant response. Accessions PI462336 and PI610825 and Cv. ‘Cashel’ showed an apparent reduction in root development during stress, but this was not statistically significant. There was, however, a statistical difference in root dry biomass between treatments for accessions PI632553 and PI223178 (Fig. 6), further indicating that clones of these accessions were more susceptible to drought than the other accessions.

### Overall response to drought stress and control conditions

The results from the preceding section were combined to determine how each accession was affected by drought stress. The responses are ranked in Table 1. Accession PI418701 was not subject to negative effects under drought stress, so the clones of this accession could be considered drought tolerant, while PI462336 was only mildly affected by drought and PI610825 had an intermediate negative response under drought stress. The following cultivars and accessions had an increasing drought-susceptible response, Cv. ‘Cashel’, PI231565, PI201187, PI611044, PI223178 and PI632553.

**Table 1.** Overall review of the results for the *in vivo* drought stress experiment. +, ‘tolerant’, i.e. no difference in response between stressed and non-stressed conditions; I, ‘intermediate’, i.e. modest difference in response between stressed and non-stressed conditions; –, ‘sensitive’, i.e. strong difference in response between stressed and non-stressed conditions. Overall response was scored by taking the average of +s, Is and –s.

Accession/cultivar	Phenotypical assessment of shoot development	Phenotypical assessment of root development	Relative water content	Root dry biomass	Overall
PI418701	+	+	I	+	+ 3
PI462336	+	+	−	I	+ 1
PI610825	−	+	I	I	0
Cv. ‘Cashel’	−	+	−	I	−1
PI231565	−	I	−	I	−2
PI201187	−	−	I	I	−2
PI611044	−	−	I	I	−2
PI223178	−	−	I	−	−3
PI632553	−	−	−	−	−4

### RNA editing evaluation

cDNA samples were randomly selected for analyses of RNA editing. A colony screen is considered a highly reliable method to determine the differences in RNA editing (Roberson and Rosenthal 2006). Results obtained from the colony screen were compared with those obtained from the trace-file method (results not shown). The highest difference observed between methods was a 10.8 % difference in editing, whereas the lowest difference observed was 0.8 %. This confirmed our confidence in the trace-file method for determining editing efficiency.

**Figure 7.**
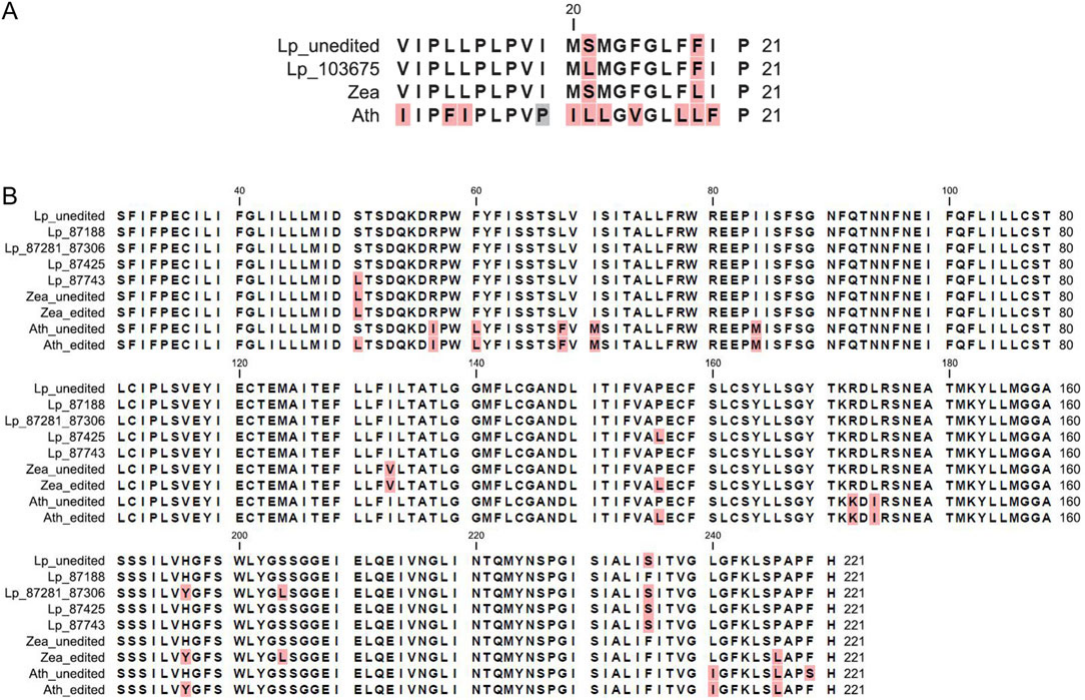
Partial peptide alignments of *ndhF* (A) and *ndhB* (B) showing the editing sites. (A) The alignment shows the *ndhF* region from amino acid 10 to amino acid 30 around the *L. perenne* editing site at amino acid 21 at nucleotide position 103675 of the *Lolium* chloroplast genome, aligned to the corresponding regions of *Zea mays* (Uniprot P46620) and *Arabidopsis thaliana* (Uniprot P 556752). *ndhF* is not edited within this region in *Zea* and *Arabidopsis*. Editing changes polar amino acid serine, also present in the *Zea* protein, against hydrophobic amino acid leucine. (B) The *ndhB* alignment includes the *L. perenne* edited and unedited sequences of *ndhB* between amino acid 30 and amino acid 250 of the protein. The labels on the left side indicate the editing positions in the *L. perenne* chloroplast genome sequence (GenBank accession AM777385). The *Lolium* sequences are aligned with the corresponding *Z. mays* and *A. thaliana* protein sequences. The *A. thaliana* chloroplast sequence is derived from GenBank accession AP000423, and the edited sites were based on Tillich *et al.* (2005). The *Z. mays* chloroplast sequence is taken from X86563, the editing sites from UniProt entry P0CD58.

All observed editing events were C-to-U changes with a polar amino acid serine to a hydrophobic leucine conversion in *ndhF* and serine-to-leucine, proline-to-leucine, histidine-to-tyrosine and serine-to-phenylalanine changes in *ndhB* (Fig. 7A and B). In addition to the serine-to-leucine change in *ndhB*, the serine-to-phenylalanine and histidine-to-tyrosine changes were changes from polar amino acids to hydrophobic amino acids. The most significant change is probably the proline-to-leucine amino acid change leading to a greater structural change and the function of the protein.

**Figure 8.**
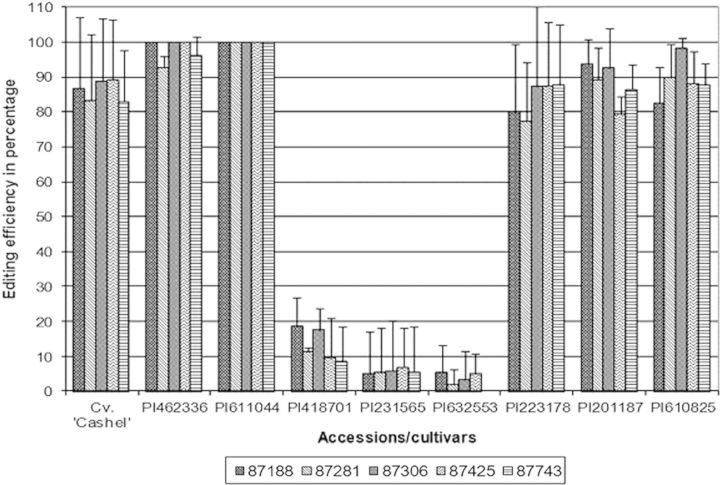
RNA editing efficiency within the *ndhB* transcript of editing sites located on plastid genome positions 87188, 87281, 87306, 87425 and 87743 (from the NCBI gene bank, chloroplast genome of *L. perenne*: http://www.ncbi.nlm.nih.gov/nuccore/AM777385) in drought-stressed plants (editing at site 87743 in P1632553 is zero). Error bars represent the standard deviation of the mean.

No statistically significant differences in RNA editing of any *ndhB* or *ndhF* transcripts were detected within accessions, between stressed and non-stressed clones, for any of the analysed editing sites (data not shown). However, there were significant and reproducible differences between accessions for editing efficiency. For the editing sites within the *ndhB* transcript, the editing efficiencies of Cv. ‘Cashel’ and accessions PI462336, PI611044, PI223178, PI201187 and PI610825 were statistically different (*P* < 0.05) in comparison with the editing efficiencies of accessions PI418701, PI231565 and PI632553 (data for the drought-stressed plants are shown in Fig. 8). The observed differences were dramatic. Some accessions showed almost complete editing, while other accessions almost completely lacked editing at these sites.

**Figure 9.**
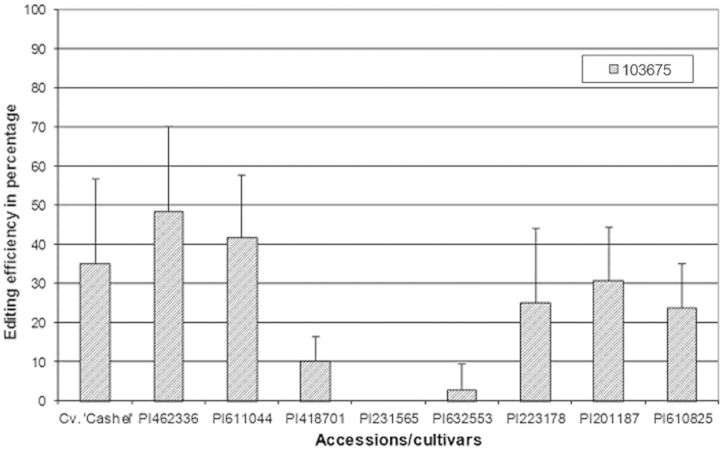
RNA editing efficiency of the editing site within the *ndhF* transcript located on plastid genome position 103675 (from the NCBI gene bank, chloroplast genome of *L. perenne*: http://www.ncbi.nlm.nih.gov/nuccore/AM777385) in drought-stressed plants (editing at site 103675 in P1231565 is zero). Error bars represent the standard deviation of the mean.

The known editing site at genome position 103675 within the *ndhF* transcript showed a similar difference between accessions as was evident for the *ndhB* editing sites; however, the editing efficiency of accessions PI223178 and PI610825 was not statistically different (*P* < 0.05) from that of accession PI418701 (data for the drought-stressed plants are shown in Fig. 9).

### Relationship between drought stress response and RNA editing efficiency within the *ndhB* and *ndhF* transcripts

There was no correlation between drought tolerance and editing efficiencies for editing sites within the *ndhB* and the *ndhF* genes (see Table 2). For example, the two most drought-tolerant accessions, PI462336 and PI418701, showed very different editing efficiencies, while the more susceptible accessions could be either efficiently or inefficiently edited.

**Table 2.** Editing efficiency in relation to drought tolerance for the tested clones of different accessions.

Accessions/cultivars (specific genotypes)	Editing efficiency		Drought stress response
	Sites within the *ndhB* gene	Sites within the *ndhF* gene	
PI418701	Low	Moderately low	Tolerant
PI462336	High	High	Tolerant
PI610825	High	Moderately high	Moderately tolerant
Cv. ‘Cashel’	High	High	Intermediate
PI231565	Low	Low	Intermediate
PI201187	High	High	Intermediate
PI611044	High	High	Intermediate
PI223178	High	Moderately high	Moderately susceptible
PI632553	Low	Low	Susceptible

## Discussion

Editing efficiencies for the editing sites within *ndhB* and *ndhF* were evaluated within the accessions tested for drought tolerance, and subsequently compared with the respective drought tolerance of these clones. These results showed dramatic differences in the editing efficiency of these transcripts between accessions, but no alteration in efficiency in response to stress or any correlation between drought tolerance and editing efficiency within these *ndh* genes. Previous reports (Casano *et al.* 2000) have demonstrated that expression levels of plastid NDH complex genes were up-regulated during drought stress (a situation which causes photo-oxidative stress) and play a role in reducing PQ in conjunction with superoxide dismutase and hydroquinone peroxidase (Casano *et al.* 2000; Abdeen *et al.* 2010). The current finding does not contradict the involvement of the NDH complex in circumventing oxidative stress, but indicates that the RNA editing of the involved transcripts is not a major determining factor for regulation of this complex. Nevertheless, very low editing efficiencies (as low as 5 %) were observed within certain accessions. This may indicate that these genes are highly up-regulated, to the extent that, despite inefficient editing, enough functional transcripts are produced to allow for correct assembly of sufficient NDH complex to counter oxidative stress.

An alternative explanation could be that another pathway is more prominently involved in countering oxidative stress. This pathway could be the PGR5/PGRL1-dependent route, also known as the non-NDH pathway (Rumeau *et al.* 2007; Suorsa *et al.* 2009). The involvement of this pathway with cyclic electron transfer was shown to be important under near-optimal conditions in *Arabidopsis* (Munekage *et al.* 2004). A recent publication showed that components of the PGR5/PGRL1 route were up-regulated during drought stress, whereas those of the NDH complex *ndhH* were not affected (Lehtimäki *et al.* 2006).

The observed differences in RNA editing efficiencies may be due to different expression levels of the proteins involved in the editing of these specific editing sites. These may be genotype specific and unrelated to environmental stimuli. Another possibility could be the difference in the amount of transcripts of *ndhB* and *ndhF* available for editing. If there are fewer transcripts available, then the editing efficiency might increase. Both these explanations could contribute to the observed effects. Other studies have identified certain *trans*-factors that are essential for editing of certain sites; however, this does not exclude the possibility that other proteins may be involved in the editing machinery, as is implied by Chateigner-Boutin *et al.* (2008). The editing machinery can be limited by the least available protein within that editing complex. This was demonstrated when chimeric RNA was expressed containing the editing site of *psbL* in tobacco chloroplasts, and this led to a significant decrease in the editing efficiency of the endogenous *psbL* RNA. This competitive effect of the transgene was specific to the *psbL* gene, with other editing sites being properly edited, indicating depletion of the *psbL*-specific *trans-*acting factor (Chaudhuri *et al.* 1995).

Some proteins that bind to specific *cis*-factors surrounding the editing sites have been identified (Okuda *et al.* 2010). These belong to the PPR protein family. This large family of proteins is believed to be involved in RNA maturation in plastids and mitochondria (Shikanai 2006). In *Arabidopsis* mitochondria, gene knockout of a PPR did result in an altered drought stress response, in this case an enhancement, but this occurred in plants severely retarded in growth as a result of impaired mitochondrial NDH activity (Yuan and Liu 2012). It is difficult to draw parallels between gene knockouts impairing NDH activity in the mitochondria, where the complex has an essential bioenergetic function, and in the chloroplasts where it has a purely adaptive role, and can be abolished without phenotypic consequence in the absence of stress (Horváth *et al.* 2000).

While PPR proteins appear to have a role specific to individual editing sites, other proteins within the editing complex might have a more general function, and if knocked out, could impair the whole editing machinery. For example, when the CP31 protein in tobacco was knocked out, editing within the *psbL* transcript was completely absent, while editing in the *ndhB* gene was partially impaired. Further elucidation of the regulation of RNA editing in perennial ryegrass plastids, and its consequences for the production of functional NDH complex, await further identification of all the *trans*-factors involved in the *ndhB* and *ndhF* genes in this species.

## Conclusions

This study shows that different varieties or accessions of crop plants can differ markedly in the extent to which plastidial transcripts are edited, with some sites, in certain accessions, being edited to very low levels. However, in the case of genes in the NDH complex, associated with the oxidative stress response, there is no evidence that RNA editing makes a significant contribution to regulation. Up-regulation of the complex, associated with drought stress, is primarily mediated through other processes, which merit further investigation.

## Sources of Funding

The work was funded by Teagasc (Ireland) through Walsh postgraduate Fellowships to Rob van den Bekerom and Kerstin Diekmann.

## Contributions by the Authors

The bulk of the experimental work described was part of the PhD thesis of the first author. Its design, execution and interpretation were heavily dependent on the parallel PhD thesis of the third author. The corresponding author was the co-supervisor of the first author and was actively involved in the execution of the experiments, while the second author was the Academic PhD supervisor of the first author. All four authors met regularly during the course of the work to discuss and monitor its progress, and all four co-operated closely in the preparation of this manuscript.

## Conflict of Interest Statement

None declared.

## References

[PLT035C1] Abdeen A, Schnell J, Miki B (2010). Transcriptome analysis reveals absence of unintended effects in drought-tolerant transgenic plants overexpressing the transcription factor ABF3. BMC Genomics.

[PLT035C2] Barrs HD, Weatherley PE (1962). A re-examination of the relative turgidity technique for estimating water deficits in leaves. Australian Journal of Biological Sciences.

[PLT035C3] Bock R, Kossel H, Maliga P (1994). Introduction of a heterologous editing site into the tobacco plastid genome: the lack of RNA editing leads to a mutant phenotype. EMBO Journal.

[PLT035C4] Burrows PA, Sazano LA, Svab Z, Maliga P, Nixon PJ (1998). Identification of a functional respiratory complex in chloroplasts through analysis of tobacco mutants containing disrupted plastid *ndh* genes. EMBO Journal.

[PLT035C5] Casano LM, Zapata JM, Martin M, Sabater B (2000). Chlororespiration and poising of cyclic electron transport: plastoquinone as electron transporter between thylakoid NADH dehydrogenase and peroxidase. Journal of Biological Chemistry.

[PLT035C6] Chateigner-Boutin AL, Ramos-Vega M, Guevara-Garcia A, Andres C, de la Luz Gutirrez-Nava M, Cantero A, Delannoy E, Jimenez LF, Lurin C, Small I, Leon P (2008). CLB19, a pentatricopeptide repeat protein required for editing of rpoA and clpP chloroplast transcripts. The Plant Journal.

[PLT035C7] Chaudhuri S, Carrer H, Maliga P (1995). Site-specific factor involved in the editing of the psbL mRNA in tobacco plastids. EMBO Journal.

[PLT035C8] Diekmann K, Hodkinson TR, Wolfe KH, Van Den Bekerom R, Dix PJ, Barth S (2009). Complete chloroplast genome sequence of a major allogamous forage species, perennial ryegrass (*Lolium perenne* L.). DNA Research.

[PLT035C9] Foito A, Byrne SL, Shepherd T, Stewart D, Barth S (2009). Transcriptional and metabolic profiles of *Lolium perenne* L. genotypes in response to a PEG-induced water stress. Plant Biotechnology Journal.

[PLT035C10] Gamborg OL, Miller RA, Ojima KK (1968). Nutrient requirements of suspension cultures of soybean root cells. Experimental Cell Research.

[PLT035C11] Gray MW (2012). Evolutionary origin of RNA editing. Biochemistry.

[PLT035C12] Holden NM, Brereton AJ (2002). An assessment of the potential impact of climate change on grass yield in Ireland over the next 100 years. Irish Journal of Agricultural and Food Research.

[PLT035C13] Horváth EM, Peter SO, Joët T, Rumeau D, Cournac L, Horváth GV, Kavanagh TA, Schafer C, Peltier G, Medgyesy P (2000). Targeted inactivation of the plastid *ndhB* gene in tobacco results in an enhanced sensitivity of photosynthesis to moderate stomatal closure. Plant Physiology.

[PLT035C14] Ibanez H, Ballester A, Munoz R, Quiles MJ (2010). Chlororespiration and tolerance to drought, heat and high illumination. Journal of Plant Physiology.

[PLT035C15] Joët T, Cournac L, Horváth EM, Medgyesy P, Peltier G (2001). Increased sensitivity of photosynthesis to antimycin A induced by inactivation of the chloroplast *nbhB* gene: evidence for a participation of the NADH-dehydrogenase complex to cyclic electron flow around photosystem I. Plant Physiology.

[PLT035C16] Kugita M, Kaneko A, Yamamoto Y, Takeya Y, Matsumoto T, Yoshinaga K (2003). The complete nucleotide sequence of the hornwort (*Anthoceros formosae*) chloroplast genome: insight into the earliest land plants. Nucleic Acids Research.

[PLT035C17] Lehtimäki N, Lintala M, Tagliavia CP, Chaves MM, Jones HG (2006). Drought stress-induced upregulation of components involved in ferredoxin-dependent cyclic electron transfer. Journal of Plant Physiology.

[PLT035C18] Michel BE, Kaufmann MR (1973). The osmotic potential of polyethylene glycol 6000. Plant Physiology.

[PLT035C19] Munekage Y, Hashimoto M, Miyake C, Tomizawa K, Endo T, Tasaka M, Shikanai T (2004). Cyclic electron flow around photosystem I is essential for photosynthesis. Nature.

[PLT035C20] Murashige T, Skoog F (1962). A revised medium for rapid growth and bioassays with tobacco cultures. Physiologia Plantarum.

[PLT035C21] Nakae A, Tanaka T, Miyake K, Hase M, Mashimo T (2008). Comparing methods of detection and quantitation of RNA editing of rat glycine receptor alpha3. International Journal of Biological Sciences.

[PLT035C22] Okuda K, Hammani K, Tanz SK, Peng L, Myouga F, Motohashi R, Shinozaki K, Small I, Shikanai T (2010). The pentatricopeptide repeat protein OTP82 is required for editing of plastid *ndhB* and *ndhG* transcripts. The Plant Journal.

[PLT035C23] Roberson LM, Rosenthal JJ (2006). An accurate fluorescent assay for quantifying the extent of RNA editing. RNA.

[PLT035C24] Rumeau D, Peltier G, Cournac L (2007). Chlororespiration and cyclic electron flow around PSI during photosynthesis and plant stress response. Plant, Cell and Environment.

[PLT035C25] Shikanai T (2006). RNA editing in plant organelles: machinery, physiological function and evolution. Cellular and Molecular Life Sciences.

[PLT035C26] Shinozaki K, Ohme M, Tanaka M, Wakasugi T, Hayashida M, Matsubayashi T, Zaita N, Chunwongse J, Obokata J, Yamaguchi-Shinozaki K, Ohto C, Torozawa K, Meng BY, Sugita M, Deno H, Kamogashira T, Yamada K, Kusuda J, Takaiwa F, Kato A, Tobdo N, Shimada H, Sugiura M (1986). The complete nucleotide sequence of the tobacco chloroplast genome: its gene organization and expression. EMBO Journal.

[PLT035C27] Suorsa M, Sirpio S, Aro EM (2009). Towards characterization of the chloroplast NAD(P)H dehydrogenase complex. Molecular Plant.

[PLT035C28] Tillich M, Funk HT, Schmitz-Linneweber C, Poltnigg P, Sabater B, Martin M, Maier RM (2005). Editing of plastid RNA in *Arabidopsis thaliana* ecotypes. The Plant Journal.

[PLT035C29] Tillich M, Lehwark P, Morton BR, Maier UG (2006). The evolution of chloroplast RNA editing. Molecular Biology and Evolution.

[PLT035C30] van den Berg L, Zeng YJ (2006). Response of South African indigenous grass species to drought stress induced by polyethylene glycol (PEG) 6000. South African Journal of Botany.

[PLT035C31] Yuan H, Liu D (2012). Functional disruption of the pentatricopeptide protein SLG1 affects mitochondrial RNA editing, plant development, and response to abiotic stresses in *Arabidopsis*. The Plant Journal.

